# Bioprocess Engineering, Transcriptome, and Intermediate Metabolite Analysis of L-Serine High-Yielding *Escherichia coli* W3110

**DOI:** 10.3390/microorganisms10101927

**Published:** 2022-09-28

**Authors:** Chenyang Wang, Qinyu Li, Peng Zhou, Xiaojia Chen, Jiping Shi, Zhijun Zhao

**Affiliations:** 1 Biorefinery Laboratory, Shanghai Advanced Research Institute, Chinese Academy of Sciences, 99 Haike Road, Shanghai 201210, China; 2 College of Life Sciences, University of Chinese Academy of Sciences, 19 Yuquan Road, Beijing 100049, China; 3 School of Life Science and Technology, ShanghaiTech University, 393 Middle Huaxia Road, Shanghai 201210, China; 4 School of Health Science and Engineering, University of Shanghai for Science and Technology, Shanghai 200093, China

**Keywords:** L-serine, *E. coli*, metabolic engineering, fermentation

## Abstract

L-serine is widely used in the food, cosmetic, and pharmaceutical industries. However, the complicated metabolic network and regulatory mechanism of L-serine production lead to the suboptimal productivity of the direct fermentation of L-serine and limits its large-scale industrial production. In this study, a high-yield L-serine production *Escherichia coli* strain was constructed by a series of defined genetic modification methodologies. First, L-serine-mediated feedback inhibition was removed and L-serine biosynthetic pathway genes (*serA^fr^*, *serC*, and *serB*) associated with phosphoglycerate kinase (*pgk*) were overexpressed. Second, the L-serine conversion pathway was further examined by introducing a *glyA* mutation (K229G) and deleting other degrading enzymes based on the deletion of initial *sdaA*. Finally, the L-serine transport system was rationally engineered to reduce uptake and accelerate L-serine export. The optimally engineered strain produced 35 g/L L-serine with a productivity of 0.98 g/L/h and a yield of 0.42 g/g glucose in a 5-L fermenter, the highest productivity and yield of L-serine from glucose reported to date. Furthermore, transcriptome and intermediate metabolite of the high-yield L-serine production *Escherichia coli* strain were analyzed. The results demonstrated the regulatory mechanism of L-serine production is delicate, and that combined metabolic and bioprocess engineering strategies for L-serine producing strains can improve the productivity and yield.

## 1. Background

L-serine (L-Ser) is a non-essential amino acid that has wide applications in the food, pharmaceutical, and cosmetic industries [1,2]. Additionally, L-Ser has been identified as one of the top 30 most interesting biochemical building blocks [3]. Currently, L-Ser production relies on enzymatic or direct fermentation [4,5], and the global L-Ser production capacity (350 tons per year) is well below the expected market demand (3000 tons per year) [6]. Therefore, it is necessary to develop a more effective L-Ser production method. Furthermore, demand exists to develop a direct fermentation approach that can be implemented with low-cost substrates, simple operational purification procedures, and a reduction in pollution, as enzymatic conversion always utilizes the expensive precursors glycine and methanol [7].

L-Ser production by microbial fermentation has been extensively studied in *Corynebacterium glutamicum*. In 2007, Petra Peters-Wendisch et al. engineered a *C. glutamicum* strain with an industrial production capacity 36 g/L L-Ser from glucose [8,9,10]. The strain was constructed by overexpressing the L-Ser pathway genes, deleting the L-Ser dehydratase *sdaA*, and reducing the expression of serine hydroxymethyltransferase (SHMT) encoded by *glyA,* and was cultured with an external folate supply [8,9,10]. The replacement of folate with corn steep liquor led to the production of an L-Ser titer of 43 g/L from sucrose in 96 h by another *C. glutamicum* strain with random mutation and minimization of by-product synthesis [11]. However, as a host, *Escherichia coli* has attracted attention due to its well-characterized genetic background, amenability to genetic manipulation, and faster growth rate, higher fermentation intensity, and better utilization rate of glucose in direct fermentation [12].

Recently, Hemanshu Mundhada et al. developed a strain of *E. coli* with a production capacity of 11.7 g/L L-Ser via ultraviolet radiation on a 1 L scale [1]. Then, this group increased the production capacity to 37 g/L by adaptive laboratory evolution [13]. Subsequently, translation initiation optimization led to the highest reported so far for L-Ser production, 50 g/L, with a yield of 0.36 g/g from glucose in 60 h [14]. However, the application of many random mutagenesis even makes the description of efficient L-Ser synthesis mechanisms difficult. Furthermore, previous studies have shown that repeated random mutation often leads to unknown mutations at some locations in the genome, together with targeted mutagenesis [15]. The impact of these unanticipated mutations is difficult to determine.

**Figure 1:**
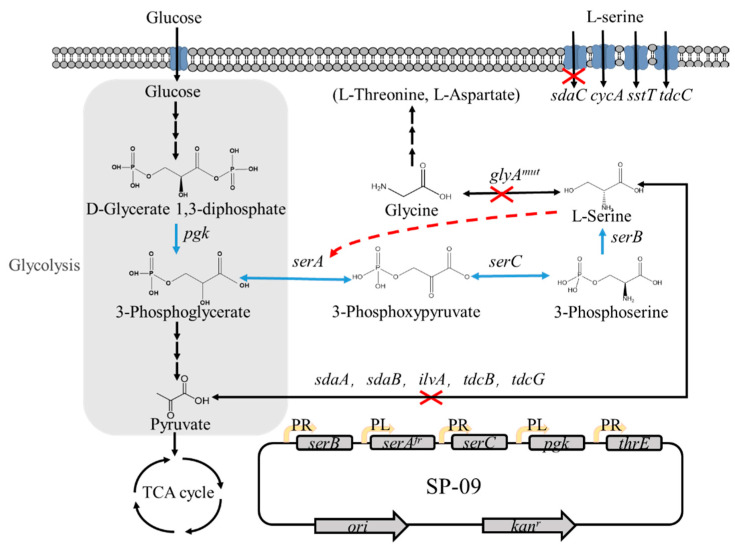
L-Serine biosynthesis and its regulation in *E. coli*. The blue arrows indicate overexpression of the relevant genes using the SP vector. The red “X” indicates the deletion of relevant genes. The red dashed line indicates feedback inhibition.

In this study, we constructed a L-Ser high-yielding strain derived from *E. coli* W3110. As shown in Figure 1, a series of genetic manipulations was introduced to enhance the biosynthesis, adjust the degradation and conversion pathway, and optimize the transporter system of L-Ser, thus improving the productivity of L-Ser and the yield of batch fermentation. Further transcriptome and intermediate metabolite analysis elucidated the regulatory mechanism of L-Ser production in *E. coli*, providing a platform for metabolic engineering design to construct L-Ser high-yielding strains.

## 2. Materials and Methods

### 2.1. Strains, Media, and Materials

Wild-type *E. coli* W3110 was used as the parent strain for serial engineering of L-Ser production. *E. coli* 5α was used for the cloning and propagation of plasmids. *E. coli* BL21(DE3) was used for the enzyme assays. Further strains constructed in this study are shown in Table 1. For strain construction, cultures were grown at 30 °C or 37 °C in Luria-Bertani medium (LB; 10 g/L tryptone, 10 g/L NaCl, 5 g/L yeast extract) and supplemented with antibiotics as appropriate.

For L-Ser production, minimal M9 medium (6.8 g/L Na_2_HPO_4_, 3 g/L KH_2_PO_4_, 0.5 g/L NaCl, 1 g/L NH_4_Cl, 0.015 g/L CaCl_2_·2H_2_O, 0.49 g/L MgSO_4_·7H_2_O, and 2.8 × 10^−4^ g/L MgSO_4_·7H_2_O) supplemented with 2 g/L yeast extract and 9 g/L glucose was used in a shake flask. Fed-batch cultures contained 3 g/L MgSO_4_·7H_2_O, 0.017 g/L CaCl_2_·2H_2_O, 1 g/L NaCl, 5 g/L (NH_4_)_2_SO_4_, 0.07 g/L FeSO_4_·7H_2_O, 0.11 g/L Na-citrate·2H_2_O, 2 g/L yeast extract, 8 g/L glucose, and 1.5 mL/L 1000× mother liquor of a composite additive of trace elements (7 g/L CoCl_2_·6H_2_O, 2.5 g/L CuSO_4_·5H_2_O, 25 g/L H_3_BO_3_, 16 g/L MnCl_2_·4H_2_O, 1.5 g/L Na_2_MoO_4_·2H_2_O, and 3 g/L ZnSO_4_·7H_2_O).

Plasmid DNA was isolated by using a Plasmid Mini-Prep Kit (BIO Basic Inc., Markham, ON, Canada). Chromosomal DNA from *E. coli* W3110 was isolated by using a Genomic DNA Isolation Kit (BIO Basic Inc.). Agarose gel purification of DNA fragments was performed using a TaKaRa Agarose Gel DNA Purification Kit Ver. 2.0 (TAKARA BIOTECHNOLOGY CO., Kusatsu, Japan). Restriction enzymes, ligases, and other DNA manipulation enzymes were used according to the manufacturer’s manuals (TAKARA BIOTECHNOLOGY CO.). All plasmid constructs were verified by DNA sequencing (BIO Basic Inc.). Plasmid DNA was transformed into competent *E. coli* cells by electroporation (Table 2).

### 2.2. Construction of Gene Knockout Mutants

Genes were knocked out singly or in combination using the method reported by Kirill A. Datsenko and Barry L. Wanner [16]. The primers used for amplification of the kanamycin cassette from the plasmid pKD4 are shown in Table S1. The helper plasmids pKD13, pKD46, and pCP20 were used for the construction of knockout mutants. All gene knockout strains were verified by sequencing with the primers shown in Table S1.

### 2.3. Construction of the glyA Mutant Library

Random mutagenesis was induced by error-prone PCR, and the mutation rate was controlled at 0.66%. The genomic DNA of *E. coli* W3110 was utilized as the template with the primers *glyA*-F/R (Table 3). PCR reagents were mixed in a volume of 50 μL according to the following composition: 10× reaction buffer, 10 pmol each primer, 2 μmol MnCl_2_, 2 μmol MgCl_2_, 1 μmol Taq DNA polymerase, and unbalanced dNTPs. PCR products were purified and digested with *Nde* I and *Hind* III and were then subcloned into the expression vector pT 7-7. BL21 (DE3) cell transformed with these expression vectors grew in LB at 30 °C. Sequentially, the *glyA* sequence in pT7-7-*glyA* was replaced with these different *glyA* mutation constructs using site-directed mutagenesis with the primers shown in Table S2.

### 2.4. Chromosomal Integration of Glymut Constructs

Pcas and PtargetF [17] were synthesized by GenScript (Nanjing, China). The sgRNA primer including the N20 sequences, followed by the protospacer adjacent motif (PAM) sequence and donor DNA primer, *glyA*-D, used in this study, are shown in Table 3. Genes were replaced using the method reported by Yu Jiang et al. [17]. All gene knockout strains were verified by sequencing.

### 2.5. Plasmid Construction for Overexpression of L-Serine Biosynthetic Pathway Components

All plasmids used for plasmid construction are described in Table 1. The low copy number vector SP is a laboratory stock plasmid and contains the temperature-sensitive lambda repressor cItS857 gene and the lambda PR and PL promoters. The vector SP was used as the backbone for all of plasmids constructed in this study. The L-Ser biosynthetic genes *serA*, *serB* and *serC* were amplified from *E. coli* W3110 using the primers shown in Table 3. The *serA^fr^* mutant was generated by mutating two residues in *serA*, His344 and Asn346, to alanines by site-directed mutagenesis with the primers shown in Table 3. *SerA*-p1 and *serA*-p2 were used to clone *serA^fr^* into the *Xba I*/*Nhe I* site in SP under the control of the PL promoter, yielding the plasmid SP-01. This plasmid was later used to clone *serC*, generating SP-02. The gene *serB* was cloned into the SP-02 vector at the *Bgl II* and *Sca I* site, generating SP-05. Subsequently, the gene *pgk*, encoding phosphoglycerate kinase, was cloned into the SP-05 vector backbone, yielding the vector SP-08. The gene *thrE*, encoding the L-Ser/L-threonine exporter, was amplified from *C. glutamicum* ATCC 13032. The resulting 1.7-kb fragment was cloned into the corresponding restriction sites in SP-08, generating in the vector SP-09.

### 2.6. PGDH and SHMT Enzyme Assays

BL21(DE3)/pT7-7-*serA^fr^* cells were harvested at the mid-exponential growth phase through centrifugation after induction by isopropyl-beta-d-thiogalactopyranoside (IPTG), and crude extracts were obtained using ultrasonication. PGDH in crude extracts was purified by ion exchange chromatography (AKTA) on a Sepharose Fast Flow column, and diethylaminoethyl dextran gel (DEAE) was used as the anion exchange agent [18]. PGDH activity was determined by the determination of α-ketoglutarate (α-KG) reductase activity instead of glyceric acid-3-phosphate dehydrogenase activity. The 1-mL reaction system contained 40 mM potassium phosphate buffer (pH = 7.5), 1.0 mM DL-dithiothreitol (DTT), 0.25 mM NADH, 5 mM α-KG, and 10–30 μg of the purified crude extract [19].

BL21(DE3)/pT7-7-*glyA^mut^* growth was induced by IPTG at an OD_600_ of 0.5, and the culture was centrifuged to obtain bacterial cells at an OD_600_ of 4. SHMT activity was determined by a continuous spectrophotometric assay using DL-3-phenylserine hydrate and phosphopyridoxal as the substrates [20]. Hydrolysis of DL-3-phenylserine hydrate by SHMT was monitored spectrophotometrically at 279 nm to assess the formation of benzaldehyde. The standard curve was generated with a benzaldehyde concentration gradient by spectrophotometry at 279 nm in the dark. The assay buffer contained 1 mg of centrifuged bacterial cells and 1 mL of substrate (50 mM DL-3-phenylserine hydrate, 30 µM phosphopyridoxal, pH = 8.0) at 37 °C. After culture at 30 °C, 4.48 g for 1 h, the assay buffer was centrifuged at 2800× *g* for 10 min, and the supernatants were evaluated at 279 nm. The production of 1 M of benzaldehyde per hour with 1 g wet weight of the cell in 1 L assay buffer was defined as one unit.

### 2.7. Shake Flask and Fed-Batch Fermentation

For shake flask studies, a single clone was first grown in 5 mL of LB for 12–14 h, and 5 mL of the culture was transferred to 100 mL of M9 medium with supplemented 2 g/L yeast extract and 9 g/L glucose for culture in a 500 mL shake flask at 30 °C and 4.48 g. Each culture was induced after 3 h by heating to 38 °C. The shake flask studies were repeated at least three times.

Fed-batch fermentation was conducted in a 5-L bioreactor (Biostat A Plus, Sartorius Stedim, Göttingen, Germany). A single clone was precultured in 50 mL of LB and shaken at 33 °C and 4.48 g for 12 to 14 h. The culture was inoculated into 2.5 L of the fermentation medium at a 1:20 (*v*/*v*) inoculum:medium ratio at an initial temperature of 33 °C. L-Ser production was induced at an OD_600_ of 20 by heating to 38 °C. The agitation, air supplementation, and feed rate were changed to maintain the dissolved oxygen (DO) concentration above 30% saturation. The pH was controlled at 6.8 using 30% (*w*/*v*) NH_3_·H_2_O. The DO-stat feeding strategy was employed to supply exhausted nutrients to the fermenter. The feeding solution contained 50% (*w*/*w*) glucose.

### 2.8. Sample Preparation and Extraction for Intermediate Metabolite Analysis

Bioreactor-grown cells were harvested at the exponential growth phase after induction. A total of 5 mL of culture was injected into the 20 mL quenching solutions (glycerol/saline, 60/40, *v*/*v*) and directly centrifuged at 16,128× *g* at −20 °C for 3 min. After the removal of the supernatant, cell pellets were resuspended by 5 mL saline and cells were collected by centrifugation at 16,128× *g* at −20 °C for 3 min. Subsequently, cell pellets were extracted three times by cold methanol, as reported previously [21]. Cell debris was removed by centrifugation for 5 min at 16,128× *g*.

### 2.9. Analytical Methods

Bacterial growth was monitored by measuring the OD_600_ in a spectrophotometer (Beckman Germany), and the glucose concentration was analyzed using an SBA sensor machine (Institute of Microbiology, Jinan, China).

The L-Ser from the fermentation solution was determined by precolumn derivatization HPLC as follows. Two hundred microliters of cell-free supernatants, 100 µL 1 M triethylamine (TEA, with acetonitrile as the solvent), and 100 µL of 0.2 M phenylisothiocyanate (PITC, with acetonitrile as the solvent) were added into a 1.5-mL microcentrifuge tube. Then, 400 µL of n-hexane was added to the tube and incubated at room temperature for 1 h. Then, the lower solution layer (200 µL) was mixed with 800 µL of deionized water and filtered through a 0.22-µm PTFE membrane filter (Hydrophilic PTFE Syringe Filter, ANPEL Laboratory Technologies Inc., Roodepoort, South Africa) [22]. Finally, the filtrates were used for HPLC analysis with a Shimadzu Separations module connected to a Shimadzu SPD-M20A detector set to 256 nm and were separated on an Agilent Extend C-18 column (250 mm × 4.6 mm, 5 µm) using 0.05 M sodium acetate (pH = 6.50 ± 0.05) (mobile phase A) and methanol:acetonitrile:water (20:60:20, *v*/*v*/*v*) (mobile phase B) with a flow rate of 0.8 mL/min, and a constant column temperature of 45 °C. The gradient time course was as follows: initial condition, A:B (93:7, *v*/*v*); 13 min, A:B (0:100, *v*/*v*); 19 min, A:B (93:7, *v*/*v*); 25 min, completed elution.

Amino acids within the cell were determined using an AB SCIEX QTRAP 5500 system with an Agilent InfinityLab Poroshell 120 HILIC-Z (2.1 mm × 100 mm, 2.7 μm) at 35 °C. Conditions of the mass spectrometer were as follows: curtain gas, 35 psi; ion source gas 1, 60 psi; ion source gas 2, 60 psi; source temperature, 550 °C; polarity, positive; ionspray voltage, 5500 V. Mobile phase A was 20 mM ammonium formate in water at pH 3 whereas mobile phase B consisted of 20 mM aqueous ammonium formate at pH 3 in acetonitrile/water (9:1, *v*/*v*). The flow rate of the mobile phase was 0.25 mL/min. The gradient time course was as follows: initial condition, A:B (5:95, *v*/*v*); 15 min, A:B (35:65, *v*/*v*); 17 min, A:B (35:65, *v*/*v*); 18 min, A:B (5:95, *v*/*v*); 20 min, completed elution.

Organic acids within the cell were determined using the AB SCIEX QTRAP 5500 system with a WATERS T3 column (4.6 mm × 150 mm, 3 μm) at 40 °C. Conditions of the mass spectrometer were as follows: curtain gas, 35 psi; ion source gas 1, 55 psi; ion source gas 2, 55 psi; source temperature, 550 °C; polarity, negative; ionspray voltage, 4500 V. Mobile phase A was 0.1% formic acid in acetonitrile whereas mobile phase B consisted of 0.1% formic acid in water. The flow rate of the mobile phase was 0.3 mL/min. The gradient time course was as follows: initial condition, A:B (2:98, *v*/*v*); 2 min, A:B (2:98, *v*/*v*); 6 min, A:B (98:2, *v*/*v*); 9 min, A:B (98:2, *v*/*v*); 9.1 min, A:B (2:98, *v*/*v*); 13.5 min, completed elution.

### 2.10. Transcriptome Datasets

For the transcriptome analysis, we only used the transcriptome datasets that were obtained at the exponential growth phase after induction. The samples were frozen immediately in liquid nitrogen and sent to Sangon Biotech (Shanghai, China) for transcriptome sequencing. Total RNA was extracted using the Bacterial Total RNA Extraction Kit (TIANGEN, Beijing, China). Sequencing was performed based on the Illumina Xten platform. Raw sequencing data were quality-controlled and mapped to the reference genomic sequences, and then the reads mapped to genes were counted. After calculating the expression of genes, differentially expressed genes (DEGs), clusters of orthologous groups (COGs), KEGG functional enrichment analysis, and KEGG pathway analysis were performed. DEGs were identified according to the following rules: a log2-fold change (FC) >2 and a *p* value < 0.05 [23].

## 3. Results and Discussion

### 3.1. Construction of the L-Serine Production Strain from E. coli W3110

**Figure 2:**
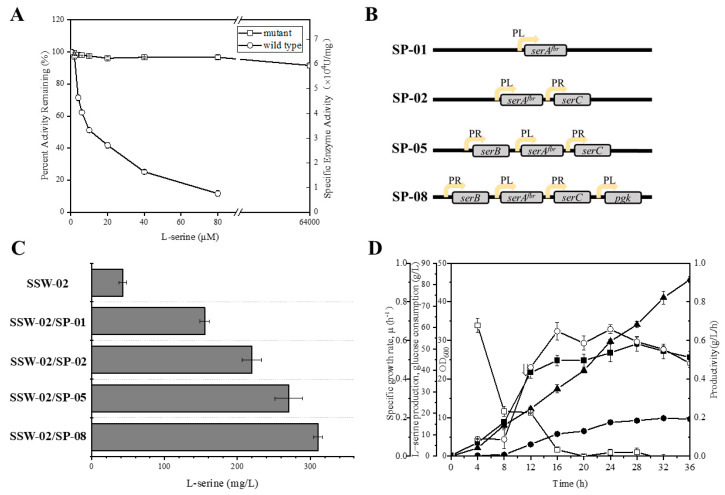
Optimization of the synthetic *serA^fr^CB* operon with *pgk* and screening for high-level L-serine production. (**A**) PGDH^mut^ (*serA^fr^*, open squares), PGDH^wt^ (*serA*, open circles). (**B**) The structure of different plasmids. The yellow arrow with PR or PL indicates PR or PL promoters. (**C**) L-serine production by shake flask fermentation with different plasmids. (**D**) L-Serine production by SSW-02/SP-08 by fed-batch fermentation. Cell growth (filled squares), L-serine concentration (filled circles), glucose consumption (upward-pointing filled triangles), specific growth rate (open squares), and L-serine productivity (open circles). The arrow indicates the starting point of induction. The data are presented as the means ± SDs from three measurements.

As a prerequisite for L-Ser production, the activity of the branch pathway leading to L-Ser biosynthesis, which involves *serA*, *serC*, and *serB*, was enhanced. PGDH, encoded by *serA*, catalyzes the initial reaction in L-Ser biosynthesis and the catalytic activity of PGDH can be regulated by feedback inhibition by L-Ser in *E. coli* [24]. The feedback inhibition was overcome by the mutation of two residues (histidine-344 and aspartic acid-346) to alanine, as previously described, which would remove the hydrogen bonds between L-Ser and the regulatory binding domain. This led to the mutated gene named *serA^fr^* [25]. The feedback resistance of the enzyme PGDH, encoded by *serA^fr^*, was investigated by overexpressing these genes in BL21(DE3) via the pT7-7 vector. The activity of *serA^fr^* could be sustained at 95% with 64 mM L-Ser, whereas the activity of the wild-type enzyme remained at only 10% (Figure 2A). Then, *serA^fr^*, *serC*, and *serB* were overexpressed in the low copy number pSC vector containing the PR or PL promoter, resulting in SP-01, SP-02, and SP-05 (Figure 2B).

To produce L-Ser, the *sdaA* gene encoding the L-Ser-specific dehydratase was first deleted from *E. coli* W3110 to construct the SSW-01 strain. Subsequent deletion of *glyA*, encoding SHMT, which converts L-Ser to glycine, resulted in the double knockout SSW-02 strain. To evaluate the L-Ser production capacity, the resulting plasmids SP-01 (SP-*serA^fr^*), SP-02 (SP-*serA^fr^C*), and SP-05(SP-*serA^fr^BC*) were transformed into SSW-02. As shown in Figure 2C, strain SSW-02/SP-01 was grown in M9-yeast medium supplemented with 50 mmol glucose, and the final concentration of L-Ser was 155 mg/L after 15 h in a shake flask. An L-Ser concentration of 220 mg/L, 42% higher than that obtained by culturing SSW-02/SP-01, was obtained by culturing SSW-02/SP-02. SSW-02/SP-05 attained the highest L-Ser concentration, 270 mg/L, which was 1.74-fold higher compared to the only overexpressing *serA^fr^*. The L-Ser accumulation profile shown in Figure 2C indicates that the production of L-Ser increased as more biosynthetic genes were overexpressed.

Furthermore, previous studies have shown that only 15% of the carbon assimilated from glucose is directed into the L-Ser biosynthetic pathway in *E. coli* [5]. Hence, SP-*serA^fr^BCpgk* (SP-08) was constructed to increase the carbon flux from glucose to L-Ser and improve L-Ser productivity via amplification of phosphoglycerate kinase encoded by *pgk* (Figure 2B). Flask culture of the recombinant SSW-02/SP-08 strain produced a final L-Ser concentration of 311 mg/L, 15% higher than that obtained by culturing SSW-02/SP-05 (Figure 2C). Thus, overexpression of *pgk* effectively improved the L-Ser production capacity of the strain. To further examine L-Ser production of SSW-02/SP-08, fed-batch fermentation was performed in a 5-L fermenter. The highest L-Ser concentration, 17.7 g/L, was observed at 32 h with a yield of 0.24 g/g from glucose (Figure 2D). It also found that the final biomass of the SSW-02/SP-08 strain was only OD_600_ = 26, so the nonideal growth renders the strain unsuitable for fed-batch fermentations. In order to overcome this, an approach was followed to increase the serine tolerance as described below.

### 3.2. Influence of Mutations in glyA on L-Serine Production and Cell Growth

A previous study showed that the attenuation of *glyA* transcription resulted in increased L-Ser accumulation, a decrease in the purine pool, poor growth, and cell elongation [26,27]. The same phenomenon was observed in this study: SSW-02 cells were elongated and exhibited unstable growth at the early stage of fermentation (Figure S1, Supplementary Materials). We reprogrammed the predominant one-carbon source metabolism with suppressed SHMT activity to increase the stability of the strains. A series of error-prone PCRs were employed to construct a *glyA* mutation library [28]. Different reductions in SHMT activity were obtained and examined by transforming the recombinant plasmids harboring *glyA^mut^* into BL21 (DE3).

**Figure 3:**
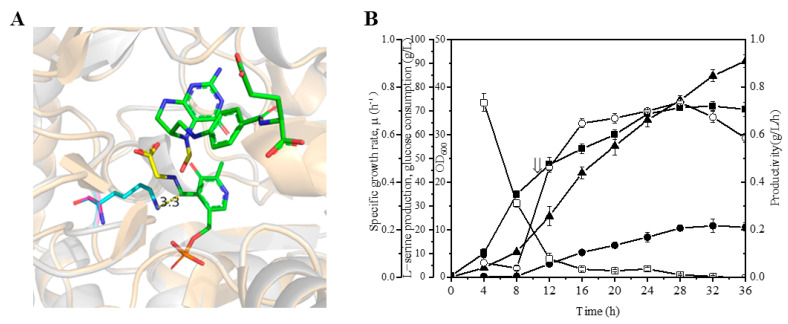
SSW-03/SP-08 L-serine production during fed-batch fermentation. (**A**) Structural modelling of SHMT mutant K229G (light gray) and comparison with the wild-type (light orange, PDB ID, 1DFO) in cartoon format. The cofactor PLP and THFA are shown as sticks in green. The L-serine degradation products glycine and formyl group are the sticks in yellow. K229 of the wild-type is shown in cyan and G229 of the mutant is in magenta. The O is in red and the N is in blue. (**B**) SSW-03/SP-08 L-serine production by fed-batch fermentation. Cell growth (filled squares), L-serine concentration (filled circles), glucose consumption (upward-pointing filled triangles), specific growth rate (open squares), and L-serine productivity (open circles). The arrow indicates the starting point of induction. The data are presented as the means ± SDs from three measurements.

As shown in Table 4, SHMT encoded by *glyA^mut^* (K229G) showed an activity of 0.13 U, which decreased by 41% compared to the wild-type. The mutant K229G was modeled by SWISSMODEL based on the wild-type SHMT (PDB ID, 1DFO). Figure 3A shows the close view of the SHMT K229G mutant compared with the wild-type SHMT complexed with cofactor PLP (pyridoxal 5′-phosphate) and THFA (PDB ID, 1DFO). The side chain of the K229, which involved the degradation of L-Ser, was removed to obtain the mutant K229G [29]. Sequentially, the *glyA* gene in SSW-01 was replaced with the appropriate *glyA^mut^*(K229G) via CRISPR/Cas9 to generate SSW-03 (Δ*sdaA glyA^mut^*). Then, the L-Ser biosynthesis plasmid SP-08 was transformed into SSW-03, and cell growth and L-Ser production were evaluated. As shown in Figure 3B, *glyA^mut^* introduction resulted in a 24% increased biomass, and the cultured cells maintained stable growth throughout repeated experiments. SSW-03/SP-08 produced 21.6 g/L of L-Ser, which increased 22% when compared to SSW-02/SP-08.

### 3.3. Influence of sdaB, ilvA, tdcB and tdcG Deletion on L-Serine Production

**Figure 4:**
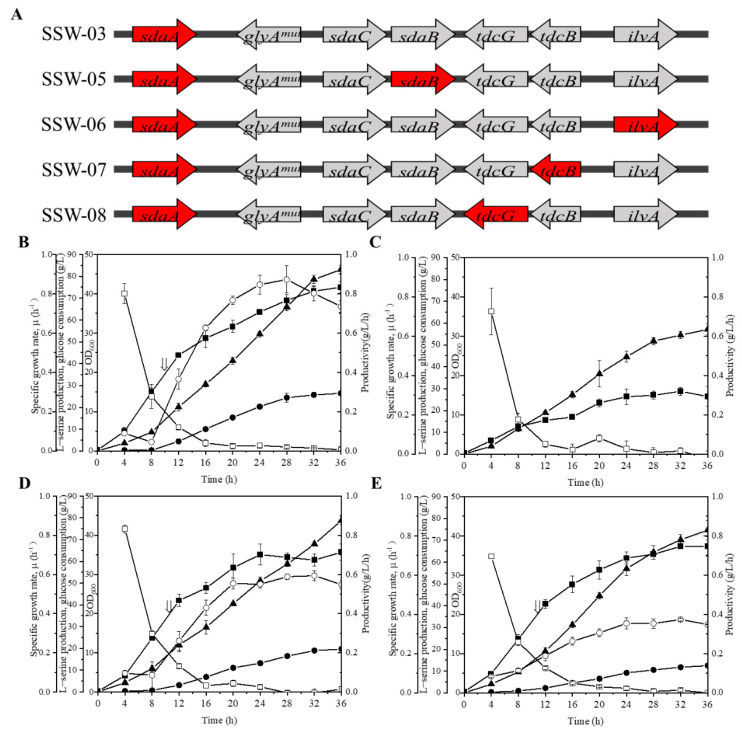
Effect of *sdaB*, *ilvA*, *tdcB*, or *tdcG* deletion on L-serine production and biomass. (**A**) Genotypes of the strains. The genes marked in red indicate targets for knockout. (**B**) L-serine production by and growth of SSW-05/SP-08 cells. (**C**) Cell density achieved by SSW-06/SP-08. (**D**) L-serine production by and growth of SSW-07/SP-08 cells. (**E**) L-serine production and cell density achieved by SSW-08/SP-08. Cell growth (filled squares), L-serine concentration (filled circles), glucose consumption (upward-pointing filled triangles), specific growth rate (open squares), and L-serine productivity (open circles). The arrow indicates the starting point of induction. The data are presented as the means ± SDs from three measurements.

The L-Ser production capacity of *E. coli* was significantly increased by the overexpression of *serA^fr^*, *serB*, *serC,* and *pgk* via knockout of the *sdaA* and mutation of *glyA*. The four genes other than *sdaA* and *glyA* (i.e., *sdaB*, *ilvA*, *tdcB*, and *tdcG*) have been reported to transform L-Ser to pyruvate in *E. coli* [30,31], but few studies have systematically investigated the individual contribution of these degradation genes to L-Ser production. To prevent the degradation and improve the production of L-Ser, *sdaB*, *ilvA*, *tdcB*, and *tdcG* were knocked out individually in the SSW-03 background to generate strains SSW-05, SSW-06, SSW-07, and SSW-08 (Figure 4A). The plasmid SP-08 was transformed into these mutant strains to produce L-Ser. As shown in Figure 4B, strain SSW-05/SP-08, which had *sdaB* deletion, showed the highest L-Ser production of 26.5 g/L, which increased 23% compared to SSW-03/SP-08. This result was expected, because the SSW-05/SP-08 biomass was also increased by 16%, and SSW-05/SP-08 showed an L-Ser productivity of nearly 0.87 g/L/h at 28 h. While the *ilvA* gene was knocked out, the growth of the strains was severely inhibited, and production could not be induced during the fermentation of SSW-07/SP-08 (Figure 4C). The growth restriction of SSW-06/SP-08 may be due to the disruption of branched chain amino acid synthesis by the deletion of *ilvA* [32]. Regarding the *tdcB* gene, the marginal difference in the L-Ser titer and biomass between the SSW-03/SP-08 and SSW-07/SP-08 strains indicated that deletion of *tdcB* is insufficient to improve L-Ser production (Figure 4D). However, the fermentation of the deletion of *tdcG* exhibited unexpected results. This *tdcG* gene knockout strain, SSW-08/SP-08, showed the same biomass as SSW-03/SP-08, but 42% lower L-Ser production than the strain (OD_600_ ~ 36, 21.6 g/L) (Figure 4B,E). The complex phenomenon associated with SSW-08/SP-08 may be caused by regulation of the expression of the interrupted operon *tdcABCDEFG* by the deletion of *tdcG*. These results suggest that only the deletion of *sdaB* improved L-Ser production, increasing the L-Ser titer by 23%; thus, SSW-05 with only the deletion of *sdaB* was selected for the following experiment, which would avoid being severely affected in cell growth by knockout of all the L-Ser dehydratases.

### 3.4. Effect of Engineering L-Serine Transport System on Strain Productivity

**Figure 5:**
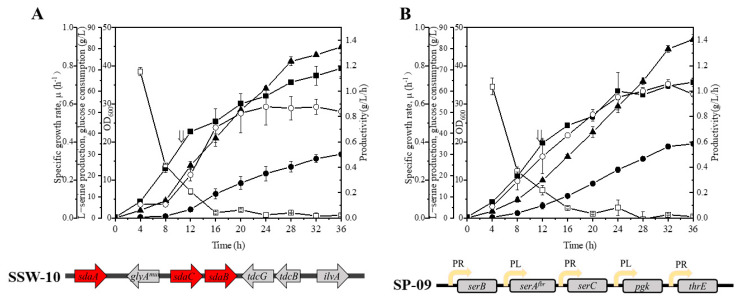
L-serine production by SSW-10/SP-08 and SSW-10/SP-09 during fed-batch fermentation. (**A**) L-serine production by and growth of SSW-011/SP-08 cells. (**B**) L-serine production and cell density achieved by SSW-10/SP-09. The genes marked in red indicate targets for knockout. The yellow arrow with PR or PL indicates PR or PL promoter. Cell growth (filled squares), L-serine concentration (filled circles), glucose consumption (upward-pointing filled triangles), specific growth rate (open squares), and L-serine productivity (open circles). The arrow indicates the starting point of induction. The data are presented as the means ± SDs from three measurements.

Moreover, the engineering amino acid transport system is also important to further improve the strain productivity by blocking the reuptake of amino acids and reducing futile cycles [33,34]. In *E. coli*, four genes, *sstT* [35], *cycA* [36], *sdaC* [37], and *tdcC* [38], have been reported to be involved in L-Ser uptake. Notably, *sdaC* is the only gene described as a highly specific L-Ser importer, and deletion of *sdaC* was found to improve L-Ser production in our recent studies [37,39]. Thus, the highly specific L-Ser uptake gene *sdaC* was deleted from SSW-05 to reduce the unwanted futile cycles caused by L-Ser reuptake; this deletion resulted in strain SSW-10. As shown in Figure 5A, the SSW-10/SP-08 produced 30 g/L L-Ser with a yield of 0.37 g/g from glucose, 16% higher than that of SSW-05/SP-08. In addition, the final L-Ser productivity of SSW-10/SP-08 was approximately 0.84 g/L/h, which was almost 1.15-fold that of SSW-05/SP-08.

Efflux pump is an important component of the amino acid transport system and it is known to increase the strain tolerance by accelerating the export of amino acids from cells. However, no research to date has reported well-characterized L-Ser exporters in *E. coli*. *ThrE* has been identified as an L-Ser/L-threonine exporter in *C. glutamicum* [40]. The *thrE* family has been identified as amino acid exporters in select bacteria, archaea, and eukaryotes, but no homologues have been found in *E.coli* [41]. Here, heterologous expression of *thrE* was performed to verify whether it worked in *E.coli*. Thus, *thrE* was cloned into the constructed expression vector SP-08 adjacent to the PR promoter, resulting in the plasmid SP-09 (Figure 5B). This recombinant plasmid was then transformed into SSW-10. Figure 5B shows the fermentation process of SSW-10/SP-09. Overexpression of *thrE* resulted in a decreased final OD_600_ value and a 16% increased L-Ser production compared to those of SSW-10/SP-08. Although the L-Ser production by the final strain SSW-10/SP-09 (35.1 g/L) was lower than L-Ser production by the strains constructed by adaptive laboratory evolution and translation initiation optimization (50 g/L), strain SSW-10/SP-09 exhibited the highest productivity (1.1 g/L/h) and yield (0.42 g/g) of L-Ser from glucose observed to date [14].

### 3.5. Transcriptomic Analysis of E. coli W3110 and SSW-10/SP-09

To investigate the effect of L-Ser fermentation on intracellular metabolism, transcriptomic analyses of *E. coli* W3110 and SSW-10/SP-09 were performed in the exponential phase. A total of 1679 transcripts were found to be significantly different under two criteria (*p*-value < 0.05 and fold change > 2.0). Transcription levels in central carbon metabolism including glycolysis, tricarboxylic acid (TCA) cycle, and amino acid pathways related L-Ser synthesis were compared.

**Figure 6:**
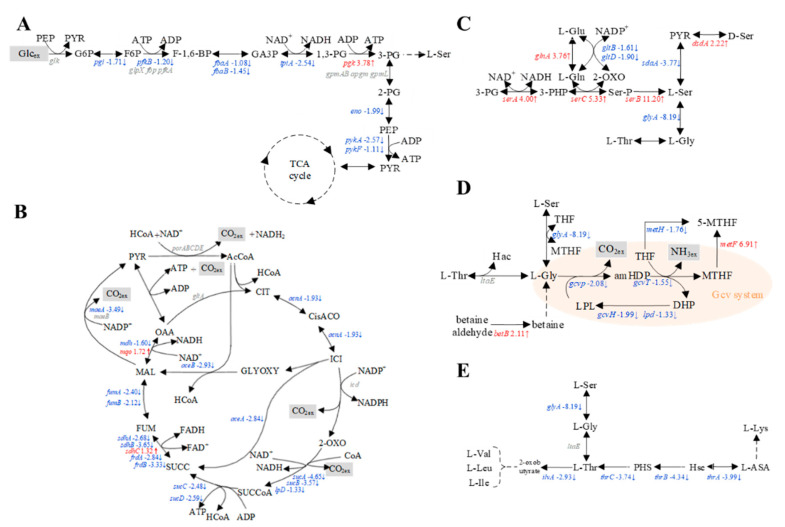
Gene expression levels related to the glycolysis, TCA cycle, and amino acid pathways related to L-Ser synthesis. (**A**) Glycolysis, (**B**) TCA cycle, (**C**) L-serine, (**D**) L-glycine, and (**E**) L-threonine biosynthesis of SSW-10/SP-09. Significant changes in the expression ratio to transcript levels are represented by color (*p*-value < 0.05, and fold changes > 2.0), three independent replicates. The red color indicates a higher expression level of SSW-10/SP-09; blue color indicates a lower expression level of SSW-10/SP-09; gray color indicates no changes; gene with number indicates log2 ratio (SSW-10/SP-09/W3110).

Expression of the genes related to most reactions in the glycolysis such as *pgi*, *fabAB*, *tpiA*, *eno*, and *pyk* was downregulated in SSW-10/SP-09, while that of *pgk*, encoding phosphoglycerate kinase, was upregulated due to its expression in plasmid SP-09 (Figure 6A). In the TCA cycle, the expression of most genes was also downregulated in SSW-10/SP-09 (Figure 6B). As the main machinery for adenosine triphosphate (ATP) synthesis, the TCA cycle could produce 12.5 ATP molecules per pyruvic acid (PYR) molecule with important intermediates such as oxaloacetate (OAA) and acetyl-CoA (AcCoA) [42]. Downregulation of the TCA cycle might cause inferior growth with less energy supply. However, the *mqo* gene encoding malate dehydrogenase that converts malate with quinone to oxaloacetate and reduced quinone was upregulated. Reduced quinone could significantly decrease the global DNA methylation level cells, and cause acute oxidative damage [43]. Reduced quinone rise in SSW-10/SP-09 may be another reason for biomass decrease. In this study, the OD_600_ of SSW-10/SP-09 was 24, a decrease of 35% compared to that of *E. coli* W3110 (OD_600_ = 37). Gene *sdhC*, encoding the succinate dehydrogenase (ubiquinone) cytochrome b560 subunit, was related to oxygen availability and upregulated in SSW-10/SP-09 [44].

Next, we analyzed changes in the expression of genes related to L-Ser production in SSW-10/SP-09 (Figure 6C). The expression levels of *serA*, *serC*, and *serB* increased in varying degrees. Expression of the gene *glnA* related to conversion from L-glutamic acid (L-Glu) to L-glutamine (L-Gln), which provided NH4^+^ for L-Ser biosynthesis, was upregulated. It caused a damping reaction in L-Glu, L-Gln, and 2-oxoglutarate (2-OXO) such as *gltB* and *gltD*. Expression of the *dsdA* encoding D-Ser ammonia-lyase was upregulated. However, the expression of *cysEKO, late,* and *trpAB* involved in L-cysteine (L-Cys) and L-tryptophan (L-Trp) biosynthesis did not change. Likewise, SSW-10/SP-09 showed the downregulation of glycine cleavage (Gcv) system genes such as *gcvT*, *gcvP*, and *gcvH* due to less intracellular glycine (Gly) (Figure 6D). This could result in a decreased number of one-carbon units and poor growth [45]. However, *metF*, encoding 5,10-CH_2_-THF reductase, involved in one-carbon metabolism drastically increased, which could compensate for the one-carbon unit [46]. Expression of the *betB* encoding the enzymes that convert betaine aldehyde to betaine was upregulated. Betaine could regulate intracellular osmotic pressure and provide methyl [47]. With supplementary betaine, the production of L-threonine, cobalamin, and L-lactate was increased [48]. The expression of genes related to the metabolism of L-threonine (L-Thr), a downstream amino acid of L-Ser, was analyzed (Figure 6E). The expression levels of *ilvA*, which was involved in both L-Thr and L-Ser dehydration, were decreased.

In addition to the above genes, there were still a large number of differentially expressed genes. Among the upregulated genes in the “pyrimidine metabolism” of SSW-10/SP-09, there were five gene sets: *rutA* (expression ratio 2^5.37^), *rutB* (expression ratio 2^4.55^), *rutC* (expression ratio 2^4.09^), *rutD* (expression ratio 2^2.30^), and *rutE* (expression ratio 2^2.44^). The rut pathway may be proposed to enhance the rate of hydrolysis of aminoacrylate, a toxic side product of L-Ser degradation [49,50]. Among the downregulated genes, genes in the category “Galactose metabolism” were enriched in SSW-10/SP-09. This category includes *gatZ* (expression ratio 2^−3.89^), *gatA* (expression ratio 2^−6.60^), *gatB* (expression ratio 2^−8.45^), *gatC* (expression ratio 2^−6.55^), *gatD* (expression ratio 2^−6.87^), and *gatR’* (expression ratio 2^−3.52^). Genes *gatZABCDR’* related to dihydroxyacetone phosphate synthesis from galactitol and galactosamine were highly involved in biofilms and downregulation of the operon may have a connection to the cell density decrease [51].

### 3.6. Intermediate Metabolite Analysis of E. coli W3110 and SSW-10/SP-09

**Figure 7:**
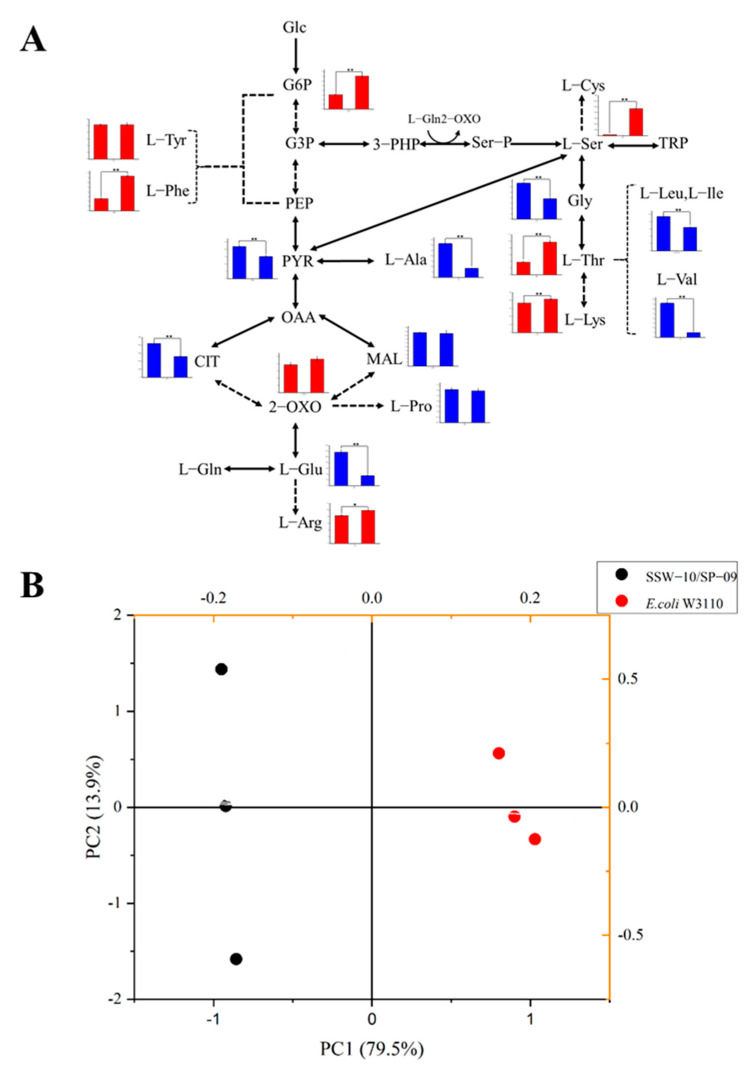
The PCA score and intracellular metabolites of SSW-10/SP-09 and *E. coli* W3110. (**A**) Seventeen intracellular metabolites of SSW-10/SP-09 and *E. coli* W3110 (three independent replicates). Significant changes in cofactors are represented by * (*p* < 0.05) and ** (*p* < 0.01). (**B**) PCA score.

As shown in Figure 7A, a set of 17 intracellular metabolites including glycolytic intermediates, intermediate metabolite in TCA cycle and amino acid related L-Ser, were measured. A score plot of the principal component analysis (PCA) model using 17 intracellular metabolites showed the discrimination of metabolite profiles depending on different strains (Figure 7B). In the PCA model, the intracellular metabolite profiles of *E. coli* W3110 and SSW-10/SP-09 were clearly discriminated. Along the axis of PC1 of the score plot, the metabolite profiles of *E. coli* W3110 were located on the positive side, while the metabolite profiles of SSW-10/SP-09 were located on the negative side.

The intracellular glucose-6-phosphate (G6P) concentration of SSW-010/SP-09 increased, which may be caused by the downregulation of most downstream genes such as *pgi*, *fabAB*, and *eno* in glycolysis (Figure 6A and Figure 7A). Intracellular PYR concentration decreased due to weak glycolysis and efficient carbon flux on L-Ser. In the TCA cycle, 2-OXO concentration and malic acid (MAL) concentration showed no significant changes between SSW-10/SP-09 and *E. coli* W3110. Intracellular L-Ser concentration was 472.5 µg/L/g, which was 32-fold of the control. Consumption of L-Gln, pitched into the second step of L-Ser biosynthesis, caused damage of its precursor L-Glu. High intracellular L-Thr concentration was in favor of maintaining L-Gly concentration [13,52]. It was also the reason for the lessened concentration of L-valine (L-Val), L-leucine (L-Leu), and L-isoleucine (L-Ile). Higher intracellular L-Thr concentration also caused the downregulation of *thrABC* (encoding homoserine dehydrogenase I, homoserine kinase, and threonine synthetase), which was consistent with the results shown in Figure 6E and Figure 7A due to its feedback inhibition [22]. The intracellular L-phenylalanine (L-Phe) concentration of SSW-10/SP-09 increased 182% when compared to that of *E. coli* W3110. However, no distinct relationship has been reported between L-Phe and L-Ser production to date.

## 4. Conclusions

L-serine is widely used in the food, cosmetic, and pharmaceutical industries, and the direct fermentation of L-serine from glucose is an attractive technique. However, L-serine producers have historically been developed via classical random mutagenesis due to the complicated metabolic network and regulatory mechanism of L-serine production, leading to un-optimal productivity and yield of L-serine, and thus limiting its large-scale industrial production.

In this study, an L-Ser-producing strain was constructed from *E. coli* W3110 by introducing a series of defined genetic manipulations. The key genes (*serA^fr^*, *serC*, *serB*, and *pgk*) for L-serine biosynthesis were overexpressed. Then, the transformation pathways were weakened by introducing a *glyA* mutation (K229G) and deleting *sdaA* and *sdaB*. Moreover, the L-Ser uptake gene *sdaC* was deleted and the L-serine/L-threonine exporter *ThrE* was overexpressed. The rational design strategies described here significantly improved the L-serine productivity and yield in fed-batch fermentation. L-serine production of 35 g/L with the highest productivity of 0.98 g/L/h and yield of 0.42 g/g glucose was finally achieved in the L-Ser high-yield strain SSW-10/SP-09.

Furthermore, the analysis of transcriptome and intermediate metabolites was performed in this study to understand the regulatory mechanisms of L-serine production. Transcription levels in central carbon metabolism including glycolysis, tricarboxylic acid (TCA) cycle, and amino acid pathways related L-Ser synthesis were compared. Expression of the genes related to most reactions in the glycolysis such as *pgi*, *fabAB*, *tpiA*, *eno*, and *pyk* was downregulated in SSW-10/SP-09. The intermediate metabolite results showed that the intracellular glucose-6-phosphate (G6P) concentration increased and intracellular PYR concentration decreased in SSW-010/SP-09. Meanwhile, there was no distinct relationship indicated between L-Phe and L-Ser production.

In summary, the fermentation-based process described herein provides an important step toward the industrial production of L-serine directly from glucose. Moreover, further strain development can be achieved through the genetic optimization of SSW-10/SP-09. This study provides basic principles for rationally designing high-yield production strains and providing a platform for metabolic engineering design to construct L-Ser high-yielding strains.

## Figures and Tables

**Table 1:** The strains used in the experiment.

Name	Description	Source
*E.coli* DH5α	Host of plasmid	Lab stock
*E.coli* BL21(DE3)	Expression strains	ATCC 26003
*E.coli* W3110	Parental strains	ATCC 23275
SSW-01	W3110 Δ*sdaA*	This work
SSW-02	W3110 Δ*sdaA glyA*	This work
SSW-03	W3110 Δ*sdaA glyA^mut^*	This work
SSW-05	W3110 Δ*sdaA glyA^mut^ sdaB*	This work
SSW-06	W3110 Δ*sdaA glyA^mut^ ilvA*	This work
SSW-07	W3110 Δ*sdaA glyA^mut^ tdcB*	This work
SSW-08	W3110 Δ*sdaA glyA^mut^ tdcG*	This work
SSW-10	W3110 Δ*sdaABC glyA^mut^*	This work

**Table 2:** The plasmids used in the experiment.

Name	Description	Source
pT 7-7	*amp* marker, T7 promoter	Takara
pSC	Low copy number, *kan* marker, p15A replicon, lambda PR and PL promoters	Lab stock
pKD13	*amp* and *kan* markers	[16]
pKD46	*amp* marker, temperature-sensitive	[16]
pCP20	*amp* and *chl* makers, temperature-sensitive	[16]
pMD19-T simple	*amp* maker, TA cloning vector, 2692 bp	Takara
Pcas	*repA101(Ts) kan* Pcas-cas9 ParaB-Red lacIq Ptrc-sgRNA-pMB1	[17]
PtargetF	*pMB1 aadA*	[17]
PtargetF-glyA	*pMB1 aadA* sgRNA-*glyA*	This work
pT-*serA*	pT 7-7 derivative, carrying *serA*	This work
pT-*serA^fr^*	pT 7-7 derivative, carrying *serA*	This work
pT-*glyA*	pT 7-7 derivative, carrying *glyA*	This work
pT-*glyA^mut^*	pT 7-7 derivative, carrying *glyA^mut^*	This work
SP-01	SP derivative, carrying *serA^fr^*	This work
SP-02	SP derivative, carrying *serA^fr^, serC*	This work
SP-05	SP derivative, carrying *serA^fr^*, *serC* and *serB*	This work
SP-08	SP derivative, carrying *serA^fr^*, *serB*, *serC* and *pgk*	This work
SP-09	SP derivative, carrying *serA^fr^*, *serB*, *serC*, *pgk* and *thrE*	This work

*kan*, Kanamycin monosulfate; *amp*, Ampicillin; *chl*, Chloramphenico.

**Table 3:** The primers used in the experiment.

Name	Sequence (5′–3′) ^a^	Restriction Enzyme
*serA*-p1	TCTAGAAAGAAGGAGATATACCATGGCAAAGGTATCGCTGGAG	*Xba I*
*serA*-p2	GAGCTCGTGAGTAAGGGTAAGGGAGGATTG	*Sac I*
*serB*-p1	AGATCTAAGAAGGAGATATACCATGCCTAACATTACCTGGTGCGACC	*Bgl II*
*serB*-p2	AGTACTGGCTGATATCGGAGAGTTTCTGGAC	*Sca I*
*serC*-p1	AGATCTAAGAAGGAGATATACCATGGCTCAAATCTTCAATTTTAG	*Bgl II*
*serC*-p2	CAGCTGTACGATCGGCTGAAAGCGTATAG	*pvu II*
*pgk*-p1	TCTAGAAAGAAGGAGATATACCATGTCTGTAATTAAGATGACCGATCTGG	*Xba I*
*pgk*-p2	GAGCTCTTGATGGAGTCAGTACCGACG	*Sac I*
*thrE*-p1	AGTACTAAGAAGGAGATATACCATGTTGAGTTTTGCGACCCTTCG	*Sca I*
*thrE*-p2	AGATCTAGATCTACATCAAAGTGACGCCGTCGAAG	*Xba I*
Site directed mutagenesis primers used for mutation of *serA*		
*serA*-p3	GATGCACATCGCAGAAGCA	
*serA*-p4	GCCCGGACGTGCTTCTGCGATGTGCATC	
*glyA* primers		
*glyA*-F	GGAATTCCATATGTTAAAGCGTGAAATGAAC	*Nde I*
*glyA*-R	CCCAAGCTTTTATGCGTAAACCGGGTAAC	*Hind III*
sgRNA-F	TGGCAACCCACTTCAGCACCACTAGTATTATACCTAGGAC	
sgRNA-R	GGTGCTGAAGTGGGTTGCCAGTTTTAGAGCTAGAAATAGC	
*glyA*-D-F	TGTCCAACAGGACCGCCTATAAAGGCCAAAAATTTTATTGTTAGCTGAGTCAGGAGATGCGGATGTTAAAGCGTGAAATGAACATTG	
*glyA*-D-R	GGCGTTCACGCCGCATCCGGCATGAACAACGAGCACATTGACAGCAAATCACCGTTTCGCTTATGCGTAAACCGGGTAACG	

^a^ The underlines indicate homology extensions of a target gene.

**Table 4:** The SHMT activity of various mutants.

Position	Sequence Change	Protein Change	SHMT Activity (U)
--	--	--	0.65 ± 0.033 ^a^
24	T → G	I8M (ATT → ATG)	0.63 ± 0.022
88	A → T	I30F (ATC → TTC)	0.34 ± 0.005
145	T → A	S49T (TCT → ACT)	0.58 ± 0.005
149	A → T	Q50L (CAG → CTG)	0.24 ± 0.02
301	G → T	A101S (GCT → TCT)	0.22 ± 0.016
483	A → T	Q161H (CAA → CAT)	0.66 ± 0.018
532	T → C	S178P (TCC → CCC)	0.36 ± 0.028
572	A → C	D191A (GAC → GCC)	0.6 ± 0.019
685/686	AA → GG	K229G (AAA → GGA)	0.13 ± 0.005
743	T → G	L248R (CTG → CGG)	0.56 ± 0.014
902	T → G	V301G (GTG → GGG)	0.57 ± 0.017
1073	T → C	V358A (GTG → GCG)	0.54 ± 0.01
1163	A → T	D388V (GAC → GTC)	0.57 ± 0.008
1195	A → T	I399F (ATC → TTC)	0.62 ± 0.032
1225	A → C	I409L (ATC → CTC)	0.44 ± 0.004
1238	A → C	Y413S (TAC → TCC)	0.59 ± 0.012

The data are presented as the means ± SDs from three measurements. ^a^ pT-*glyA* was used as the positive control.

## Data Availability

Not applicable.

## Supplementary Materials

The following supporting information can be downloaded at: https://www.mdpi.com/article/10.3390/microorganisms10101927/s1, Figure S1: Microscopic observation of SSW-01 and SSW-02. Table S1: Gene knockout primers used in the experiment. Table S2: Primers used for *glyA* mutation used in the experiment.

## Abbreviations

Glc_ex_	Extracellular glucose
PEP	Phosphoenolpyruvate
PYR	Pyruvic acid
G6P	Glucose-6-phosphate
F6P	Fructose-6-phosphate
F-1	Fructose-1
6-BP	6-bisphosphate
GA3P	Glyceraldehyde-3-phosphate
3-BG	3-bisphosphoglycerate
3-PG	3-phosphoglycerate
2-PG	2-phosphoglycerate
AcCoA	Acetyl-coa
Cit	Citric acid
cis-Aco	Cis-aconitic acid
ICit	Isocitric acid
2-OXO	2-oxoglutarate
SucCoA	Succinyl-coa
Suc	Succinic acid
Fum	Fumaric acid
Mal	Malic acid
OAA	Oxaloacetic acid
NAD^+^	Oxidized nicotinamide adenine dinucleotide
3-PHP	3-phosphonooxypyruvate
L-Ser	L-serine
D-Ser	D-serine
L-Leu	L-leucine
L-Ile	L-isoleucine
L-Val	L-valine
Gly	Glycine
L-Gln	L-glutamine
L-Glu	L-glutamate
PLP	Pyridoxal 5-phosphate monohydrate
THF	Tetrahydrofolate
MTHF	5,10-methylene tetrahydrofolate
LPL	Lipoylprotein
DHP	Dihydrolipoylprotein
amDHP	Aminomethyldihydrolipoylprotein
