# Supplementary material for: Bioprocess Engineering, Transcriptome, and Intermediate Metabolite Analysis of L-Serine High-Yielding Escherichia coli W3110
Source: Microorganisms. 2022 Sep 28;10(10):1927. doi: 10.3390/microorganisms10101927 (PMC9612172; doi:10.3390/microorganisms10101927)
Supplement: Supplementary file 1 [file microorganisms-10-01927-s001.zip › microorganisms-1916624-supplementary.pdf]

microorganisms

Article

Bioprocess Engineering, Transcriptome, and Intermediate Metabolite Analysis of L-Serine High-Yielding *Escherichia coli* W3110

Chenyang Wang [1,2,†], Qinyu Li [1,2,3,†], Peng Zhou [4], Xiaojia Chen [1,2,3], Jiping Shi [1,2,3,] and Zhijun Zhao [1,2,*]

1 Biorefinery Laboratory, Shanghai Advanced Research Institute, Chinese Academy of Sciences, 99 Haike Road, Shanghai 201210, China
2 College of Life Sciences, University of Chinese Academy of Sciences, 19 Yuquan Road, Beijing 100049, China
3 School of Life Science and Technology, ShanghaiTech University, 393 Middle Huaxia Road, Shanghai 201210, China
4 School of Health Science and Engineering, University of Shanghai for Science and Technology, Shanghai 200093, China
* Correspondence: shijp@sari.ac.cn (J.S.); zhaozj@sari.ac.cn (Z.Z.); Tel.: +86-138-1763-8015 (J.S.); +86-180-1628-6015 (Z.Z.)
† These authors contributed equally to this work.

Supplementary Materials

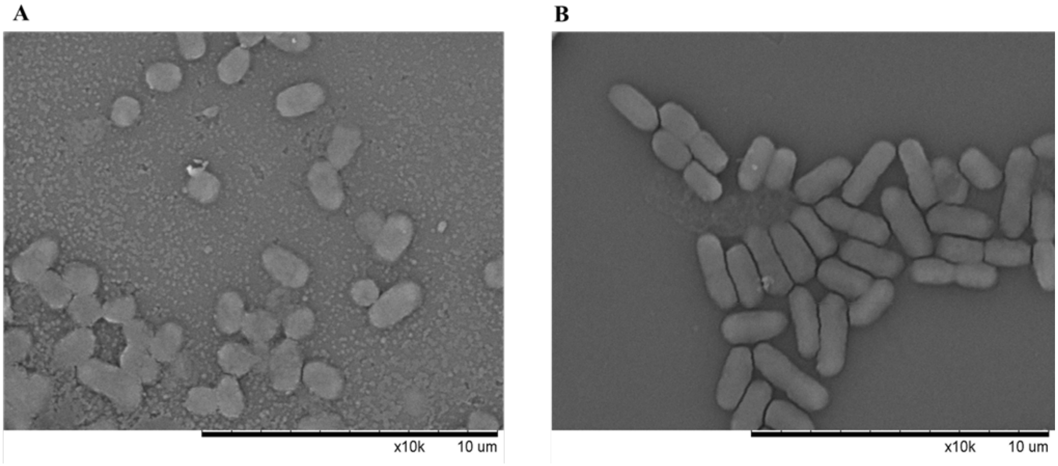

Figure S1. Microscopic observation of (A) SSW-01 and (B) SSW-02. SSW-01 cells were shorter than SSW-02 cells.

Table S1. Gene knockout primers used in the experiment.

Primer	Sequence(5′-3′)
K1	CAGTCATAGCCGAATAGCCT
K2	CGGTGCCCTGAATGAACTGC
*sdaA*_v1	CAGGCATTACATCTGGGTCGTTATCACC
*sdaA*_v2	GGTGCAGGAAGTTCAGCCAGAATGTC
*glyA*_v1	GGGTTATGAGTAAACATACGG
*glyA*_v2	CCGGTTAGTACTCAACTTGATCC
*sdaB*_v1	GATGGTGCTGACCGTAATGTTCTTCGTC
*sdaB*_v2	CTTCCAGACTCTGAAACTCGACCAGCAG
*ilvA*_v1	GTTGTCGCCGGAGATGTGGTAGTAATTC
*ilvA*_v2	GTTTTCGATGCTGGAGAATCTGGCAGTAG
*tdcB*_v1	GATCAGCGACAAAGAAATCAGCACCAG

*tdcB*_v2	GCAGGTTTCTATGTATGAAGCGCAACTG
*tdcG*_v1	GAGATCGCCACCAGAATGCAAATCAGTAC
*tdcG*_v2	GGATCTCGTACACCTTCTCAATCGTTCCTG
sdaC-v1	GAAGTCGGTATTCGCGCCATTGAAGAG
sdaC-v2	GATCGGCTGCTGAACTGTACGGATAAG

Gene-v1/K1 and gene-v2/K2 were used for colony PCR verification.

Table S2. Primers used for *glyA* mutation used in the experiment.

Primer	Sequence(5′-3′)
8-F	GAACATGGCCGATTATGATGCCGAACTGTGGC
30-F	AAGAGCACTTCGAACTGATCGCCTCCGAAAAC
49-F	GGTACTCAGCTGACCAACAAATATGCTGAAGG
50-F	GGTTCTCTGCTGACCAACAAATATGCTGAAGG
101-F	CTCCCAGTCTAACTTTGCGGTCTACACCGCGCTG
161-F	TGCCAAAGAACACAAGCCGAAAATGATTATCG
178-F	TTCTCTGCATATCCCGGCGTGGTGGACTGGGC
191-F	AAATCGCTGCCAGCATCGGTGCTTACCTGTTC
229-F	TACTACCACCACTCACGGAACCCTGGC
248-F	CGAAGAGCGGTACAAAAAACTGAACTCTGCCGT
301-F	TGGTAGAAGGGTTCCTCGAGCGCGGCTACAAA
358-F	TTTGCGACCTCCGGTATTCGTGTAGGTACTCC
388-F	ATGTGTGTCGTGCTGGACAGCATCAATGATGA
399-F	TGAAGCCGTTTTCGAGCGCATCAAAGGTAAAG
409-F	AAGTTCTCGACCTCTGCGCACGTTACCCGGTT
413-F	TTCCCCGGTTTACGCATAAAAGCTTATCATCG
8-R	CATAATCGGCCATGTTCATTTCACGCTTTAACATCA
30-R	CAGTTCGAAGTGCTCTTCCTGACGTACTTTTTCC
49-R	TTGGTCAGCTGAGTACCCTGCGCCTGCATTACG
50-R	TTGGTCAGCAGAGAACCCTGCGCCTGCATTAC
101-R	CCGCAAAGTTAGACTGGGAGCCGGAGTGCG
161-R	GCTTGTGTTCTTTGGCATGTTTTTCCAGATCGGCGTAG
178-R	CCGGGATATGCAGAGAAACCACCGATAATCAT
191-R	GATGCTGGCAGCGATTTCACGCATTTTCGCCC
229-R	ACCCGCCAGGGTTCCGTGAGTGGTG
248-R	TTTTGTACCGCTCTTCGCTACCACCTTTCGCC
301-R	GAGGAACCCTTCTACCATCGCTTTAGCGTTTT
358-R	ATACCGGAGGTCGCAAACGGGCTCTTCGGATC
388-R	TCCAGCACGACACACATCCAGCCAGCCAGTTC
399-R	GCTCGAAAACGGCTTCATCATTGATGCTGTCC
409-R	GCAGAGGTCGAGAACTTTACCTTTGATGCGCT
413-R	ATGCGTAAACCGGGGAACGTGCGCAGATGTCGAG

Ep-PCR primers used in Mutant Library Construction of *glyA*

glyA-ep-F	GGAATTCCAT ATGTTAAAGCGTGAAATGAAC
glyA-ep-R	CCCAAGCTT TTATGCGTAAACCGGGTAAC
